# *Necator americanus* Infection: A Possible Cause of Altered Dendritic Cell Differentiation and Eosinophil Profile in Chronically Infected Individuals

**DOI:** 10.1371/journal.pntd.0000399

**Published:** 2009-03-24

**Authors:** Ricardo T. Fujiwara, Guilherme G. L. Cançado, Paula A. Freitas, Helton C. Santiago, Cristiano Lara Massara, Omar dos Santos Carvalho, Rodrigo Corrêa-Oliveira, Stefan M. Geiger, Jeffrey Bethony

**Affiliations:** 1 Laboratory of Cellular and Molecular Immunology, Instituto René Rachou, Oswaldo Cruz Foundation, Belo Horizonte, Minas Gerais, Brazil; 2 Department of Microbiology, Immunology and Tropical Medicine, The George Washington University, Washington, D.C., United States of America; 3 Department of Parasitology, Federal University of Minas Gerais, Belo Horizonte, Minas Gerais, Brazil; 4 Laboratory of Helminthology and Medical Malacology, Instituto René Rachou, Oswaldo Cruz Foundation, Belo Horizonte, Minas Gerais, Brazil; Leiden University Medical Center, The Netherlands

## Abstract

**Background:**

Hookworms survive for several years (5 to 7 years) in the host lumen, inducing a robust but largely ineffective immune response. Among the most striking aspects of the immune response to hookworm (as with many other helminths) is the ablation of parasite-specific T cell proliferative response (hyporesponsiveness). While the role of the adaptive immune response in human helminth infection has been well investigated, the role of the innate immune responses (e.g., dendritic cells and eosinophils) has received less attention and remains to be clearly elucidated.

**Methodology/Principal Findings:**

We report on the differentiation/maturation of host dendritic cells in vitro and the eosinophil activation/function associated with human hookworm infection. Mature DCs (mDCs) from *Necator americanus (Necator)*–infected individuals showed an impaired differentiation process compared to the mDCs of non-infected individuals, as evidenced by the differential expression of CD11c and CD14. These same hookworm-infected individuals also presented significantly down-regulated expression of CD86, CD1a, HLA-ABC, and HLA-DR. The lower expression of co-stimulatory and antigen presentation molecules by hookworm-infected–derived mDCs was further evidenced by their reduced ability to induce cell proliferation. We also showed that this alternative DC differentiation is partially induced by excreted-secreted hookworm products. Conversely, eosinophils from the same individuals showed a highly activated status, with an upregulation of major cell surface markers. Antigen-pulsed eosinophils from *N. americanus*–infected individuals induced significant cell proliferation of autologous PBMCs, when compared to non-infected individuals.

**Conclusion:**

Chronic *N. americanus* infection alters the host's innate immune response, resulting in a possible modulation of the maturation process of DCs, a functional change that may diminish their ability for antigen presentation and thus contribute to the ablation of the parasite-specific T cell proliferative response. Interestingly, a concomitant upregulation of the major cell surface markers of eosinophils was observed in hookworm-infected individuals, indicative of antigen-specific immune responses, especially antigen presentation. We showed that in addition to the postulated role of the eosinophils as effector cells against helminth infection, activated cells may also be recruited to sites of inflammation and contribute to the immune response acting as antigen presenting cells.

## Author Summary

Hookworms survive for several years in the host lumen, inducing a robust but ineffective immune response. While the role of the adaptive response in human helminth infection has been well investigated, the role of the innate immune responses remains to be elucidated. We report on the development of dendritic cells (DCs) and the role of eosinophils during human hookworm infection. DCs from hookworm-infected individuals did not mature in the same manner as DCs from non-infected volunteers. Additionally, hookworm-infected individuals have lower expression of costimulatory (CD86) and antigen presenting molecules (CD1a, HLA-ABC, HLA-DR), which was coincident with a reduced ability of the DCs to induce cell proliferation. We also showed that this alternative DC differentiation is partially induced by excreted-secreted hookworm products. Conversely, eosinophils from the same individuals showed a highly activated status, with an upregulation of major cell surface markers. Moreover, eosinophils from hookworm-infected individuals induced a significant cell proliferation to crude antigen extracts compared to non-infected individuals. We show that, while hookworm infection modulates the development of DCs, the major cell surface markers of eosinophils are upregulated. Our data suggest that hookworm infection may alter the host's innate immune response, resulting in parasite-impaired DCs and activated, antigen presenting eosinophils.

## Introduction

Human hookworm infection is caused by the blood-feeding nematodes *Ancylostoma duodenale* and *Necator americanus*, which infects nearly 740 million people, mostly in rural areas of the tropics [1], resulting in an estimated annual loss of 22 million disability-adjusted life years [2]. These DALYs are the result of a well-established relationship between the intensity of hookworm infection, intestinal blood loss, and anemia [3]–[5]. While treatment with the benzimidazole class of anthelmintic drugs is highly effective against established hookworm infection, sustained chemotherapy has proven difficult to implement, especially in developing countries, where there is rapid reinfection (often within 12 months) [6].

Hookworms survive for several years (5 to 7 years) in the face of a robust but largely ineffective immune response. The fact that the immune system is capable of reacting vigorously to hookworm infection and yet does little to prevent primary infection or re-infection is a strong indication that the immune response to hookworms is highly down-regulated. Among the most striking aspects of this downregulation is the ablation of parasite specific T cell proliferative responses (“hyporesponsiveness”) [7]–[11]. The mechanisms underlying T cell hyporesponsiveness during helminth infections vary from organism to organism, and are associated with such diverse factors such as regulatory cytokines [12],[13], altered function of antigen presenting cells [11], [14]–[17], T cell apoptosis [18],[19], inducible NO synthase [8], modulation by regulatory T cells [20], and pro- and anti-inflammatory cytokines [11].

Marked eosinophilia is another striking feature of hookworm infection [21],[22]. As with other helminth infections, eosinophils are considered end-stage cells involved in host “protection” against hookworms [23]–[25], based on their ability to mediate antibody- (or complement-) dependent cytotoxicity *in vitro*, as well as the observation that eosinophils aggregate and degranulate in the vicinity of damaged parasites [26]. During helminth-infection, eosinophils of humans and experimental laboratory animals exhibit morphological and functional changes associated with activation in vitro [27]. These include decreased density, upregulation of surface activation molecules (e.g., CD69, CD25, CD44, and HLA-DR), enhanced cellular cytotoxicity, and release of granule proteins, cytokines, leukotrienes, and other mediators of inflammation (reviewed in [25]). Despite their potent to kill helminth parasites in vitro, the precise function of eosinophils during helminth infection remains poorly understood [25].

While the role of the adaptive immune response in human helminth infection has been well-described, the influence of the innate immune response, especially the roles of dendritic cells and eosinophils remains to be elucidated. In the current study, we report on the differentiation/maturation of dendritic cells in vitro and eosinophil activation/function associated with human *Necator* infection. We show that along with modulation in the dendritic cell maturation, possibly mediated by excreted-secreted hookworm products, there is a concomitant upregulation of the major cell surface markers on eosinophils, which is indicative of antigen-specific immune response, especially antigen-presenting cells (APCs). We suggest that chronic *Necator* infection alters the host's innate immune response, resulting in parasite-impaired dendritic cells and activated, antigen-presenting eosinophils.

## Materials and Methods

### Study population

The study was conducted in areas for endemic *N. americanus* in Northeast Minas Gerais State, Brazil. Seventeen volunteers between ages of 22 and 63, were recruited over the course of four months (Table 1). These volunteers reside in areas of high *N. americanus* transmission and presented with moderate (up to 3,999 epg) to high (>4,000 epg) intensity of *Necator* infection. Individuals were selected on the basis of not having any other helminth infection (mono-infected). The presence of *Necator* infection was determined by formalin-ether sedimentation from 2 days of fecal exams. If positive, stool samples were further examined by the Kato-Katz fecal thick-smear technique, with the intensity of infection expressed as eggs per gram of feces (epg) [28]. Six hookworm-naive individuals were initially enrolled as non-infected individuals from Belo Horizonte, Minas Gerais State, Brazil where no transmission occurs. Later, additional five hookworm-naive volunteers were included in order to demonstrate the effects of excreted-secreted hookworm products on DC differentiation. None of these individuals had a history of *Necator* infection and all presented with egg-negative stool and no specific antibodies to *Necator* crude antigen extracts. Furthermore, the nutritional status of non-infected volunteers (controls) was similar to those presented by hookworm-infected individuals as determined by anthropometric measurements. The nutritional status of adults was determined using the absolute body mass index and classified as eutrophic (18.5–24.9 kg/m^2^), underweight (<18.5 kg/m^2^) or overweight (≥25 kg/m^2^) [29],[30]. The study was approved by the Ethical Committee of Instituto René Rachou/FIOCRUZ (Protocol CEPSH/CPqRR #04/2006). Written informed consent was obtained from all participants enrolled in this study.

**Table 1:** Description of the study population.

	Individuals		Reference Values††
	*Necator*-infected	Non-infected	
Age mean, years (range)	47.2 (35.4–58.9)	36.43 (24.6–48.3)	N/A
Intensity of infection†	2181 (912–2181)	0	N/A
Hemoglobin (g/dL)	14.16 (13.3–15.0)	15.32 (13.8–16.8)	12.0–17.5
Whole blood count (cell/mm^3^)	8150 (7020–9280)	7060 (5115–9005)	3500–10000
Eosinophils (cell/mm^3^)	1008 (671–1344)*	130.6 (36–224)	50–500
% Eosinophils	12.5 (8.6–16.4)*	1.8 (0.8–2.8)	1.0–6.0

**†:** Intensity of infection was expressed by mean (range) of number of eggs per gram of feces.

**††:** Reference values for healthy adults (adapted from Elin, 2004 [51]).

*: Statistically different from control group (P<0.05).

### Monocyte isolation, generation, and maturation of human dendritic cells in vitro

Peripheral blood mononuclear cells (PBMCs) were isolated from heparinized blood by a density gradient (Histopaque 1.077, Sigma Aldrich Co., USA). Monocytes were sorted using anti-CD14-labelled magnetic beads (CD14 MicroBeads, Miltenyi Biotech Inc., USA), according to the manufacturer's instructions and were cultured in complete RPMI 1640 medium (Invitrogen Co., USA) supplemented with 2 mM of L-glutamine (Sigma), 5% heat-inactivated human AB serum (Sigma) and 6% Antibiotic-Antimycotic solution (Invitrogen). Recombinant IL-4 and GM-CSF (both from PeproTech, USA) were added to the culture at 50 ng/mL on days 1, 3 and 5. For DC maturation, cells were stimulated with 10 µg/mL of *Salmonella* lipopolysaccharide (LPS, Sigma) for 48 hours. Matured DC (mDCs) were harvested on day 7 of culture, washed twice with PBS, and used for flow cytometric analysis and other functional studies.

In order to determine the influence of hookworm antigens on the expression of mDC surface markers, monocyte-derived dendritic cells were obtained from five healthy non-exposed individuals and differentiated in the presence of *N. americanus* larval extract (L3), excreted-secreted products from adult worm (ESAw), and excreted-secreted products from L3 larvae (ESL3), obtained as previously described [9]. These antigens were added on days 1, 3 and 5 of culture at the concentration of 5 µg/mL. DC maturation was induced with LPS for 48 hours, as described above. Matured DC were harvested on day 7 of culture, washed twice with PBS, and used for flow cytometric analysis.

### Flow cytometric analysis of dendritic cells

Matured dendritic cells were stained using monoclonal antibodies to determine the expression of antigen-presentation molecules (HLA-DR, HLA-ABC and CD1a), co-stimulatory molecules (CD86, CD80 and CD40), and other monocyte markers (CD14, CD11c and CD16). Monoclonal antibodies to CD14 and CD11c were used to determine the maturation of monocyte-derived dendritic cells, with antibodies against CD16 used as a marker for expression of immunoglobulin receptor (FcγRIII). The following conjugation of monoclonal antibodies (all from BD Pharmingen, USA) was used: fluorescein isothiocyanate (FITC)-conjugated mouse anti-human CD80 (clone BB1), phycoerithrin (PE)-conjugated mouse anti-human HLA-ABC (clone DX17), CD86 (clone IT2.2), CD40 (clone 5C3), CD16 (clone 3G8), CD11c (clone B-ly6) and CD14 (clone M5E2), PE-Cy5-conjugated mouse anti-human CD1a (clone HI149) and HLA-DR (clone TU36).

Dendritic cells were harvested, washed in PBS, and then stained with antibodies at room temperature for 20 minutes. Stained cells were analyzed using a FACScan cytometer (Beckton Dickinson, USA) and CellQuest software (Becton Dickinson, USA). The intensity of fluorescence was evaluated by analysis of histograms generated by 10,000 viable cells.

### Mixed leukocyte reaction

Matured DCs (5,000) were co-incubated with 5×10^5^ heterologous PBMCs (dilution 1∶100) in 96-well flat-bottom microplates (NUNC, USA). Supernatant from cultures were collected after 5 days of culture to determine cytokine production. Thymidine incorporation was measured after 5 days of culture at 37°C and with 5%CO_2_ in a humidified incubator. After 18 hours, the cultures were pulsed with 1 µCi of [^3^H]-thymidine (Amersham Biosciences, USA). PBMCs were then harvested onto glass fiber filters, with radioactive incorporation determined by liquid scintillation spectrometry. Proliferative responses were expressed as mean counts per minute (cpm) of triplicate cultures.

### Flow cytometric analysis of eosinophils

Monoclonal antibodies were used to determine the expression of antigen-presenting molecules (HLA-DR and HLA-ABC), co-stimulatory/inhibitory molecules (CD4, CD86, CD80, CD28 and CTLA-4), activation/memory markers (CD69, CD11c, CD25, CD62LL, CD45RO and CD45RA), immunoglobulin receptors (CD64 - FcγRI, CD16 - FcγRIII, CD23 - FcεRII and CD89 - FcαRI) and Eotaxin receptor (CCR3). The monoclonal antibodies (all from BD Pharmingen, USA) used for flow cytometric analysis of eosinophils were as follows: FITC-conjugated anti human CD4 (clone RPA-T4), CD64 (clone 10.1), CD80 (clone BB1), CD28 (clone CD28.2), PE-conjugated anti-human HLA-ABC (clone DX17), CD89 (clone A59), CD86 (clone IT2.2), CD45RA (clone HI100), CD45RO (clone UCHL1), CD11c (clone B-ly6), CD62L (clone Dreg 56), CD23 (clone M-L233), CD16 (clone 3G8) or CCR3 (clone 5E8), and PE-Cy5-conjugated anti-human HLA-DR (clone TU36), CD25 (clone M-A251), CD69 (clone FN50), CTLA-4/CD152 (clone BNI3). Phenotyping of eosinophils was performed using whole blood samples. In short, 100 µL of whole blood was stained with the respective antibodies for 30 minutes at room temperature and then incubated with BD FACS Lysing Solution; unlysed cells were washed twice with PBS and then fixed. Data on fluorescently labeled cells were acquired in a FACScan flow cytometer (Becton Dickinson, USA), by gating on the eosinophil population according to Carulli et al. [31]. Intensity of fluorescence was evaluated by analysis of histograms generated by 30,000 viable cells. Isotype control antibodies (from all three fluorochromes used) were included in all experiments.

### Antigen-presentation assay using eosinophils

Eosinophils were purified from a polymorphonuclear cell (PMNCs) fraction generated after PBMC isolation. Briefly, the PMNC fraction was lysed with distillated water for 30 seconds, washed twice in PBS, and separated using a magnetic-based cell separation kit (Human Eosinophil Enrichment Kit, StemSep Technologies, USA) according to the manufacturer's instructions. Purified eosinophils were counted and cultured with complete RPMI 1640 medium in polypropylene round-bottom tubes (Becton Dickinson Labware, USA). Cells were incubated in the presence or absence of 20 third-stage (L3) *Necator americanus* larvae for 48 hours. After incubation, eosinophils were harvested, washed twice in PBS, and then fixed with fixative solution (10.0 g/L paraformaldehyde; 10.2 g/L cacodylic acid; 6.65 g/L sodium chloride; pH 7.2).

Stimulated or unstimulated eosinophils were co-incubated with autologous PBMCs in complete RPMI 1640 medium for 5 days at 37 °C and 5% CO_2_ atmosphere. Additional culture controls with PBMCs, stimulated eosinophils, and unstimulated eosinophils individually were also included. All tests were done in triplicate in 96-well flat-bottomed culture microplates. Cells were pulsed for the last 6 hours of incubation, with 1 µCi of [^3^H]-thymidine (Amersham Biosciences, USA) and harvested onto glass fiber filters. Radioactive incorporation was determined by liquid scintillation spectrometry. Proliferative responses were expressed as Stimulation index, calculated as follows: mean cpm of stimulated eosinophils and PBMC co-cultures (triplicates) divided by mean cpm of unstimulated eosinophils and PBMC co-cultures (also in triplicates).

Supernatants from eosinophil cultures were collected for determination of cytokine production after hookworm L3 stimulation and after incubation with PBMCs.

### Determination of cytokine and chemokine production by ELISA

All cytokines were detected and quantified in culture supernatants using cytokine-specific enzyme-linked immunosorbent assays kits. IFN-γ, TNF-α, IL-1β, IL-4, IL-5, IL-6, IL-10, IL-13 kits (R&D Systems, USA) were used to detect cytokine production in supernatants from the mixed leukocyte assay. IFN-γ, TNF-α, IL-4, IL-5, IL-10, IL-13, TARC/CCL17 and Eotaxin/CCL11 kits (R&D Systems, USA) were used to detect cytokine/chemokine production in cultures from eosinophils. Assays were performed according to the manufacturer's instructions. Biotin-conjugated secondary antibodies were used, followed by streptavidin-HRP (Amersham Biosciences, USA), and OPD substrate system (Sigma). The colorimetric reaction was read in an automated ELISA microplate reader at 492 nm. Calculations of cytokine/chemokine concentrations from mean optical density values were determined by interpolating diluted values from 4-parameter model fitted by SOFTmax Pro 4.8. Results were expressed in pg/mL, with the detection limits as follows: 7.8 pg/mL for IFN-γ, TNF-α and Eotaxin/CCL11; 3.9 pg/mL for IL-1β and TARC/CCL17; 15.6 pg/mL for IL-4; 11.7 pg/mL for IL-5; 4.7 pg/mL for IL-6; 23.4 pg/mL for IL-10; and 40 pg/mL for IL-13.

### Statistical analysis

The Mann-Whitney test was used to determine the differences (P value<0.05) of non-parametric variables (e.g., surface cell markers, cell proliferation, and antigen presentation) between *Necator*-infected individuals and non-infected individuals. All statistics were performed using Prism 4.0b for Macintosh (GraphPad Software, Inc.).

## Results

### *N. americanus* infection impairs dendritic cell differentiation

**Figure 1:**
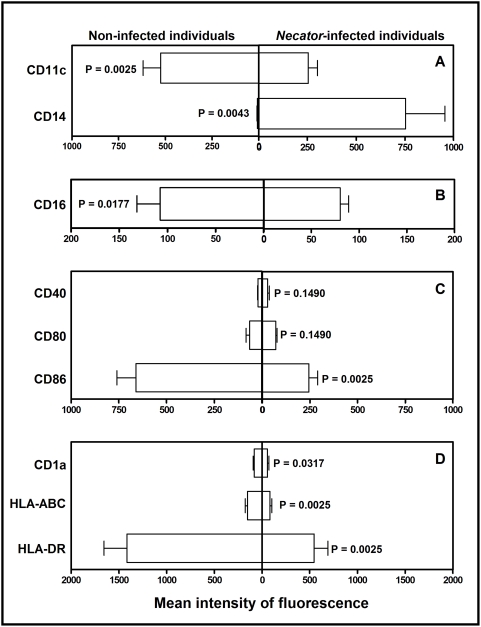
Flow cytometric analysis of monocyte-derived dendritic cell surface markers. (A) Analysis of dendritic cell differentiation/maturation (CD11c and CD14) and (B) expression of IgG receptor (CD16, FcγRIII). (C) Expression of co-stimulatory molecules. (D) Expression of cell presentation molecules. Median intensity of fluorescence is indicated on x axis (arbitrary units). Statistical differences are indicated in each graph with the significant P values.

Analysis of surface cell markers of monocyte-derived dendritic cells showed that DCs from *Necator*-infected individuals had an impaired differentiation process, as evidenced by the differential expression of CD11c and CD14 on the cell surface (**Fig. 1A**) compared to non-infected individuals. Differentiation of the monocytes into dendritic cells in non-infected individuals occurred as expected, with a relatively higher expression of CD11c and an absence lack of CD14 (**Fig. 1A**). However, dendritic cells from *Necator*-infected individuals showed a significantly lower expression of the immunoglobulin receptor CD16 (FcγRIII, P = 0.0177, **Fig. 1B**), the co-stimulatory molecule CD86 (P = 0.0025, **Fig. 1C**), and cell presentation molecules, such as CD1a (P = 0.0317), HLA-A, B, C and HLA-DR (P = 0.025 for both, **Fig. 1D**).

**Figure 2:**
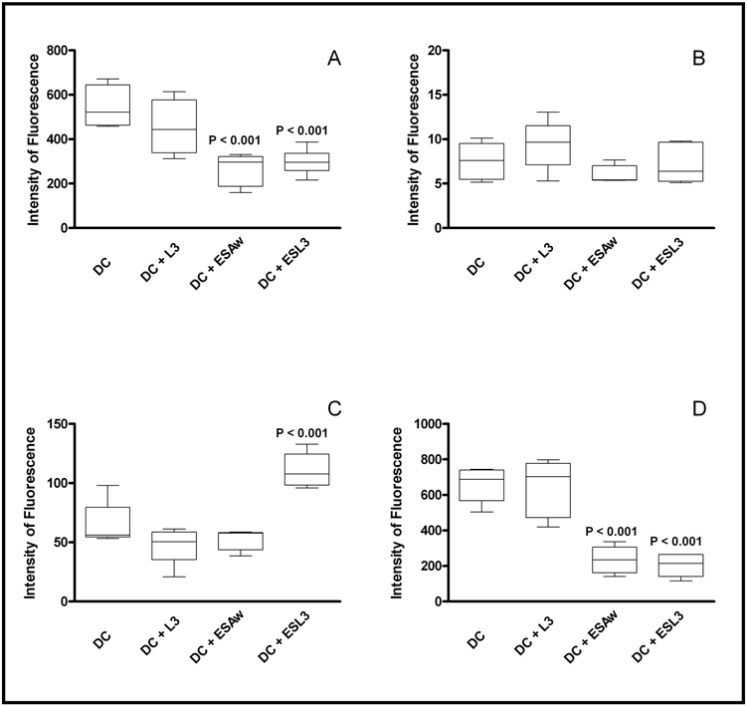
Expression of cell surface markers on monocyte-derived dendritic cells obtained from healthy non-exposed individuals (n = 5), differentiated in the presence of hookworm antigens. Expression of CD11c (A), CD14 (B), CD80 (C) and CD86 (D). Median intensity of fluorescence is indicated on y axis (arbitrary units). Statistical differences are indicated in each graph with the significant P values.

Monocyte-derived dendritic cells differentiated in the presence of *N. americanus* excreted-secreted products both from adult worm (ESAw) and L3 larvae (ESL3) showed a significant decreased expression of CD11c and CD86 while presented higher expression of CD80 (**Fig 2**). Interestingly, levels of CD14 expressed by dendritic cells differentiated in the presence of hookworm antigens were similar to those presented by control cells (**Fig. 2B**).

### Decreased cell reactivity by mixed leukocyte response

**Figure 3:**
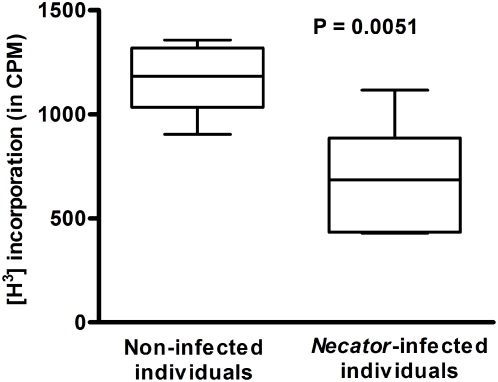
Mixed leukocyte response induced by co-culturing of dendritic cells and heterologous PBMCs. Results are expressed in counts per minute (CPM) and the bars represent the median for each group. Statistical difference is indicated with the significant P value.

In order to assess the effect of the reduced expression of CD86 and antigen presentation molecules on dendritic cells from *Necator*-infected patients, co-incubation with heterologous PBMCs was performed as a mixed leukocyte response. Due to the differences in the Major Histocompatibility Complex (MHC) marked cellular proliferation as a rejection response are expected, as observed in the co-cultures with dendritic cells from the control group (non-infected individuals) (**Fig. 3**). Our results showed that co-cultures with dendritic cells from *Necator*-infected patients resulted in markedly ablated cell proliferation (P = 0.0051) compared to non-infected individuals. This ablation may be the result of an accompanying lower expression of co-stimulatory and antigen presentation molecules on the cells of these patients.

### Phenotypic changes in the expression of eosinophil surface markers on *Necator*-infected patients

**Figure 4:**
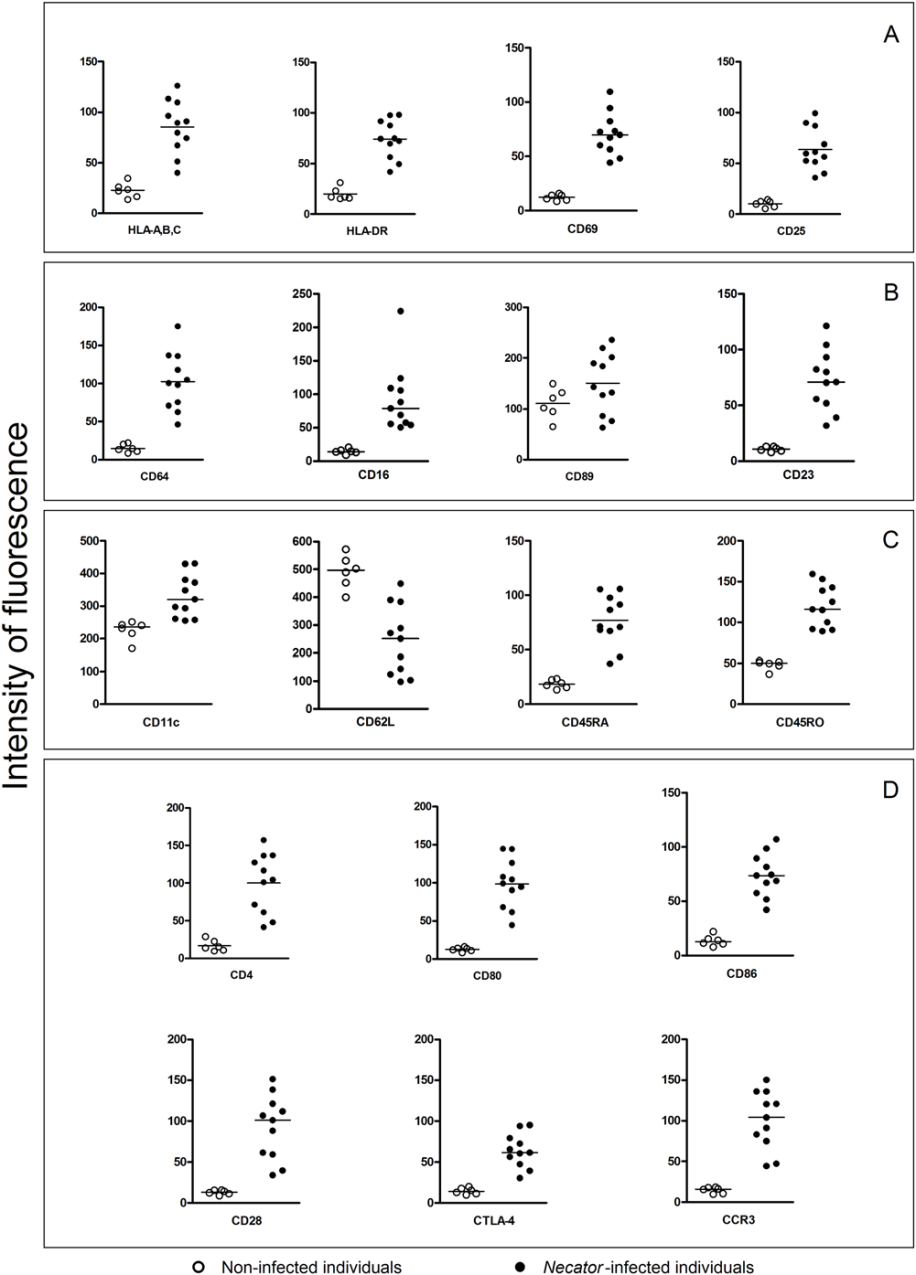
Expression of surface cell markers on eosinophils from *Necator*-infected (closed circle) and non-infected (open circle) individuals. Expression of antigen presentation/activation molecules (A), immunoglobulin receptors (B), integrin/memory markers (C) and accessory molecules/eotaxin receptor (D). Results are expressed in median intensity of fluorescence as indicated on y axis (arbitrary units). Statistically significant differences (P≤0.05) between *Necator*-infected and non-infected individuals were found for all cell surface markers tested with exception for CD89.

While there was a marked downregulation surface marker expression on dendritic cells from *Necator*-infected individuals, circulating eosinophils from these same subjects showed upregulated expression of major cell surface markers. We observed a statistically significant increase in expression of cell presentation molecules HLA-A,B,C and HLA-DR (P = 0.0011 for both), activation markers CD69 and CD25 (P = 0.0011 and P = 0.0001, respectively), naive/memory markers CD45RA and CD45RO (P≤0.0001 for both), immunoglobulin receptors (CD64 - FcγRI, CD16 - FcγRIII and CD23 - FcεRII, P = 0.0011 for all), integrin CD11c (P≤0.0001), accessory molecules (CD4, CD80, CD86, CD28 and CD152; P = 0.0011 for all), and eotaxin receptor (CCR3, P = 0.0001) (**Fig. 4**). On the other hand, the expression of CD62L, an adhesion molecule, was significantly reduced on eosinophils from *Necator*-infected individuals when compared with the non-infected individuals (P = 0.0016).

### Cell presentation in human *Necator*-infection by eosinophils

**Figure 5:**
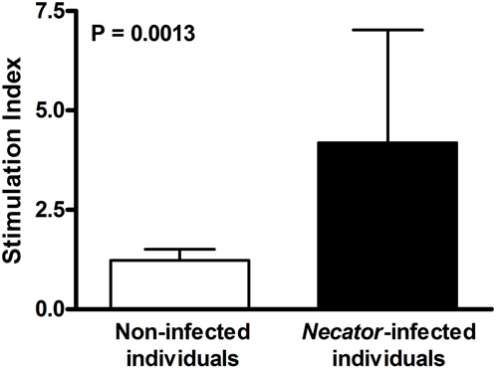
Cell proliferation response of autologous PBMCs after exposure with hookworm stimulated eosinophils from *Necator*-infected and non-infected individuals. Results are expressed as stimulation index and the bars represent the mean for each group. Statistical difference between both groups is indicated with the significant P value.

Antigen-pulsed eosinophils from *Necator*-infected individuals induced significant cell proliferation in autologous PBMCs (SI = 4.183±2.838), when compared with eosinophils from non-infected individuals (SI = 1.226±0.280, P = 0.0013, **Fig. 5**). Proliferative responses of control cultures were similar to those observed in co-cultures of primed eosinophils and PBMCs from non-infected individuals (data not shown).

### Cytokine and chemokine production by dendritic cells and eosinophils

No statistically significant differences were observed in the cytokine and chemokine production of dendritic cells and eosinophils from *Necator*-infected and non-infected individuals (data not shown). The cytokine/chemokine production of cultures from both groups was marginal and reached levels close to the detection limits of the assay for each analyte.

## Discussion

Chronic human *N. americanus* infection is associated with a profound ablation of cell proliferation that may even extend to other parasitic infections and even mitogens (“bystander effect”) [12],[32]. The mechanisms underlying *Necator*-induced T cell hyporesponsiveness have yet to be fully elucidated; however, diverse factors such as regulatory cytokines (e.g., IL-10) [12], secretion of IFN-γ by NK cells [33],[34], cleavage of effector cell chemo-attractants [35], direct down-modulation by parasite antigens [9], and reduced expression of Toll-like receptors [36],[37] have been proposed as possible causes of hyporesponsiveness. Among the many mechanisms hypothesized to cause hyporesponsiveness, the least investigated is an alteration in the antigen presenting ability of dendritic cells (DCs), which play a central role in the initiation of both innate and adaptive immune responses to parasitic helminth infection. Common functions of DCs are antigen-processing and T-lymphocyte activation, followed by changes of their surface markers, migration patterns, and cytokine production according to the different stages of parasitic infection. In vitro monocyte differentiation systems can mimic the physiological conditions that control this process in vivo. As such, they have proven useful tools for studying the factors that control DC differentiation, providing information about the physiological situations in which this process takes place [38].

The present study demonstrates that *Necator*-infection impairs phenotypic differentiation and maturation of monocyte-derived DCs, mediated by hookworm ES products, and further inhibits DCs stimulatory function to allogeneic T cell proliferation in vitro. In both murine and human systems, monocytes cultured with GM-CSF and IL-4 differentiate into immature DCs, which are characterized by low expression of MHC class II (MHC II) molecules as well as co-stimulatory molecules [38]. Immature monocyte-derived DCs can be subsequently matured with LPS, which determines the upregulation of their antigen-presenting and co-stimulatory molecules, together with higher expression of the integrin CD11c and lack of CD14. While this is indeed the case for matured DCs from non-infected donors, mDCs from *Necator*-infected individuals have a down-regulated expression of antigen-presenting, costimulatory (CD86) molecules, and CD11c, with the CD14 still present on the cell surface. The effects of *N. americanus* infection on DC function and subsequent T cell proliferation could be mediated by the down-regulated expression of antigen-presenting molecules and CD86, which may ultimately result in lower T cell specific priming. Many factors have been shown to influence the differentiation and development of DCs from their precursor cells, including corticosteroids [39], anti-inflammatory drugs [40], IFN-α and IFN-β [41], and parasitic infectious products/agents (e.g., *Plasmodium vivax* [42], *P. falciparum* [43], *Brugia malayi* [16] and *Schistosoma mansoni* [44]). The mechanism by which *Necator*-infection modulates monocyte differentiation into DCs remains unclear. However, here we demonstrated that hookworm ES products may be partially responsible for the alternative differentiation of DC, as observed by the reduction of CD11c and CD86 expression and increase of CD80 expression on the DC surface. Interestingly, the recent identification by our group of ES products from *N. americanus* (e.g., Natural Killer Cell Binding Protein (NKBP)) indicates that selective proteins released by the parasite may binds to specific cell subsets, such as NK cells, platelets, and also monocytes [34]. The identification of these proteins may shed light on this potentially unique strategy by *N. americanus* to subvert the immune response. The down-modulated DC differentiation/maturation induced during *Necator*-infection might contribute to the cellular hyporesponsiveness observed in individuals chronically infected with *N. americanus* and the long-term survival of the parasite.

Interestingly, while hookworm-infected individuals presented impaired DCs, there was marked upregulation of their circulating eosinophils. Infection with *N. americanus* is well known to be accompanied by eosinophilia, which can either be localized (e.g., to the lungs [45],[46] or the sites of attachment in the gut) or systemic [46]. The marked elevation in eosinophils during parasitic helminth infection has long been postulated to play an important role in defending the host against infection. In this case, eosinophils are thought to mediate antibody- (or complement-) dependent cellular toxicity against helminths in vitro, as they aggregate and degranulate in the local vicinity of damaged parasites in vivo [23]. Other evidence for the role of eosinophils in helminth infection are from experimentally infected mice that have been depleted of eosinophils by IL-5 neutralization and/or gene targeting and the observation that eosinophil levels increase after they are infected with helminths (reviewed in [23]). Despite these findings, the in vivo role of eosinophils in immunity to helminth infection has been much more difficult to define.

Our data show that eosinophils from *Necator*-infected individuals presented a differential expression of antigen-presenting-, costimulatory-, activation-, and memory-surface cell markers. Immunoglobulin and eotaxin receptors were also found to be upregulated. The elevated expression of antigen-presenting and co-stimulatory molecules, but reduced levels of CD62L, indicate a highly activated state for these eosinophils. Similarly, increased expression of MHC II, CD86, and CD69 molecules, but decreased CD62L levels, have also been observed in activated eosinophils after in vitro exposure to *Strongyloides stercolaris* antigens in a murine model [47]. Moreover, comprehensive analysis of human eosinophils after in vitro activation with IL-5 and GM-CSF have also showed a similar phenotypic profile (reviewed in [27]). It is noteworthy that the increased expression of CD64/FcγRI, CD16/FcγRIII and CD23/FcεRII may be directly associated with the antibody-dependent cellular toxicity against *N. americanus*. Indeed, *Necator*-infection is characterized by antibody responses dominated by IgG1, IgG4, and IgE [21], which bind to the receptors upregulated on hookworm-activated eosinophils. Surprisingly, an increased expression of CTLA-4/CD152 was observed in eosinophils from infected donors. CTLA-4 directly competes with CD28 for binding CD80/CD86 (B7) and also directs the assembly of inhibitory signaling complexes that lead to cell quiescence or anergy [48]. Further experiments are needed to elucidate its role on eosinophils during hookworm infection. Moreover, we have demonstrated that activated eosinophils express relatively higher levels of the eotaxin receptor (CCR3), suggesting that these cells may be recruited to sites of inflammation. Although the parasite has developed the ability to specifically cleave eotaxin, hypothetically inhibiting eosinophil recruitment [35], the upregulation of CCR3 expression may counterbalance this effect.

We also performed in vitro experiments to investigate whether these activated eosinophils could act as APCs during *Necator*-infection and stimulate T cell responses. Indeed, recent clinical and experimental investigations have shown that eosinophils can function as APCs [23],[47],[49]. Here we showed that, when pulsed with crude hookworm antigen extracts, eosinophils from *Necator*-infected individuals were able to initiate a specific immune response, as demonstrated by increased cellular proliferation of primed lymphocytes. As eosinophils are associated with helminth parasites at the initial stage of infection, it is possible that these cells capture antigens from helminths, migrate to T cell–rich regions, and present antigens to T cells to initiate antigen-specific immune responses. These results suggest that in addition to their role as terminal effector cells in helminth infections, eosinophils may also act as specific antigen-presenting cells. However, it is not clear yet whether activated eosinophils could compensate the lack of dendritic cell-induced response and revert, at least partially, the classic hyporesponsiveness induced by the parasite.

In summary, our work indicates that chronic *N. americanus* infection alters the host's innate immune response, resulting in parasite-impaired dendritic cells and activated eosinophils. Whereas the interaction between dendritic cells and eosinophils have been previously suggested by several authors [50]–[52], this association is not clear yet in hookworm infection. In fact, human dendritic cells may induce eosinophil chemotaxis by secreting an arachidonic acid metabolite [51]. Eosinophils may also contribute to DC maturation through eosinophil-derived major basic protein (MBP) [50]. On the other hand, the release of histamine by activated eosinophils may downregulate DC differentiation through induction of IL-10 production by lymphocytes and mDCs, after interaction with histamine receptor type 2 (HR2) on these cells [53]. Indeed, HR2 agonists acts as a suppressive molecule for antigen presentation capacity, while enhancing IL-10 production and IL-10-producing T cells [54],[55].

Based on the observation that DCs are required for development and maintenance of chronic eosinophilic airway inflammation [56],[57], the study of the eosinophil and DC interaction opens new strategies for targeting key factors to interfere with an eosinophil induced/enhanced state of diseases, such as asthma, atopic dermatitis and helminthic infections [50]. Whether this profile of host's innate immune response in *N. americanus* infection will provide further benefit to parasite survival through modulation of required adaptive responses remains to be elucidated. In the present study, we evaluated the profile of cellular components associated with the innate immune response against hookworm infection, comparing samples from hookworm mono-infected individuals living in endemic areas and healthy non-exposed volunteers. Egg-negative individuals from endemic areas were not included as controls, since the limited sensitivity of fecal exams and the long pre-patency period make it difficult to assure that the volunteers were not infected. Moreover, it has been previously shown that the immunological status of helminth-infected patients remains unaltered after anthelmintic treatment for several months [12],[58]. Although all individuals enrolled in our study received fully medical support and were matched by nutritional status, we cannot exclude the influence of other factors such as viral and bacterial infections. Our results should be further validated by large immunoepidemiological surveys in endemic areas.

## References

[1] de Silva NR, Brooker S, Hotez PJ, Montresor A, Engels D (2003). Soil-transmitted helminth infections: updating the global picture.. Trends Parasitol.

[2] Chan MS (1997). The global burden of intestinal nematode infections–fifty years on.. Parasitol Today.

[3] Brooker S, Peshu N, Warn PA, Mosobo M, Guyatt HL (1999). The epidemiology of hookworm infection and its contribution to anaemia among pre-school children on the Kenyan coast.. Trans R Soc Trop Med Hyg.

[4] Lwambo NJ, Bundy DA, Medley GF (1992). A new approach to morbidity risk assessment in hookworm endemic communities.. Epidemiol Infect.

[5] Stoltzfus RJ, Dreyfuss ML, Chwaya HM, Albonico M (1997). Hookworm control as a strategy to prevent iron deficiency.. Nutr Rev.

[6] Albonico M, Crompton DW, Savioli L (1999). Control strategies for human intestinal nematode infections.. Adv Parasitol.

[7] Candolfi E, Hunter CA, Remington JS (1994). Mitogen- and antigen-specific proliferation of T cells in murine toxoplasmosis is inhibited by reactive nitrogen intermediates.. Infect Immun.

[8] Dai WJ, Gottstein B (1999). Nitric oxide-mediated immunosuppression following murine Echinococcus multilocularis infection.. Immunology.

[9] Geiger SM, Caldas IR, Mc Glone BE, Campi-Azevedo AC, De Oliveira LM (2007). Stage-specific immune responses in human Necator americanus infection.. Parasite Immunol.

[10] Schleifer KW, Mansfield JM (1993). Suppressor macrophages in African trypanosomiasis inhibit T cell proliferative responses by nitric oxide and prostaglandins.. J Immunol.

[11] Semnani RT, Liu AY, Sabzevari H, Kubofcik J, Zhou J (2003). Brugia malayi microfilariae induce cell death in human dendritic cells, inhibit their ability to make IL-12 and IL-10, and reduce their capacity to activate CD4+ T cells.. J Immunol.

[12] Geiger SM, Massara CL, Bethony J, Soboslay PT, Correa-Oliveira R (2004). Cellular responses and cytokine production in post-treatment hookworm patients from an endemic area in Brazil.. Clin Exp Immunol.

[13] King CL, Mahanty S, Kumaraswami V, Abrams JS, Regunathan J (1993). Cytokine control of parasite-specific anergy in human lymphatic filariasis. Preferential induction of a regulatory T helper type 2 lymphocyte subset.. J Clin Invest.

[14] Loke P, MacDonald AS, Robb A, Maizels RM, Allen JE (2000). Alternatively activated macrophages induced by nematode infection inhibit proliferation via cell-to-cell contact.. Eur J Immunol.

[15] Semnani RT, Law M, Kubofcik J, Nutman TB (2004). Filaria-induced immune evasion: suppression by the infective stage of Brugia malayi at the earliest host-parasite interface.. J Immunol.

[16] Semnani RT, Sabzevari H, Iyer R, Nutman TB (2001). Filarial antigens impair the function of human dendritic cells during differentiation.. Infect Immun.

[17] Whelan M, Harnett MM, Houston KM, Patel V, Harnett W (2000). A filarial nematode-secreted product signals dendritic cells to acquire a phenotype that drives development of Th2 cells.. J Immunol.

[18] Chow SC, Brown A, Pritchard D (2000). The human hookworm pathogen Necator americanus induces apoptosis in T lymphocytes.. Parasite Immunol.

[19] Jenson JS, O'Connor R, Osborne J, Devaney E (2002). Infection with Brugia microfilariae induces apoptosis of CD4(+) T lymphocytes: a mechanism of immune unresponsiveness in filariasis.. Eur J Immunol.

[20] Taylor MD, LeGoff L, Harris A, Malone E, Allen JE (2005). Removal of regulatory T cell activity reverses hyporesponsiveness and leads to filarial parasite clearance in vivo.. J Immunol.

[21] Loukas A, Prociv P (2001). Immune responses in hookworm infections.. Clin Microbiol Rev.

[22] Ovington KS, Behm CA (1997). The enigmatic eosinophil: investigation of the biological role of eosinophils in parasitic helminth infection.. Mem Inst Oswaldo Cruz.

[23] Rothenberg ME, Hogan SP (2006). The eosinophil.. Annu Rev Immunol.

[24] Taliaferro WR, Sarles MP (1939). The cellular reactions in the skin, lungs, and intestine of normal and immune rats after infection with *Nippostrongylus brasiliensis*.. J Infect Dis.

[25] Klion AD, Nutman TB (2004). The role of eosinophils in host defense against helminth parasites.. J Allergy Clin Immunol.

[26] Desakorn V, Suntharasamai P, Pukrittayakamee S, Migasena S, Bunnag D (1987). Adherence of human eosinophils to infective filariform larvae of Necator americanus in vitro.. Southeast Asian J Trop Med Public Health.

[27] Bochner BS (2000). Systemic activation of basophils and eosinophils: markers and consequences.. J Allergy Clin Immunol.

[28] Katz N, Chaves A, Pellegrino J (1972). A simple device for quantitative stool thick-smear technique in Schistosomiasis mansoni.. Rev Inst Med Trop Sao Paulo.

[29] WHO (1995). Physical status: the use and interpretation of anthropometry.

[30] Jardim-Botelho A, Brooker S, Geiger SM, Fleming F, Lopes ACS (2008). Age patterns in undernutrition and helminth infection in a rural area of Brazil: associations with ascariasis and hookworm.. Trop Med Int Health.

[31] Carulli G, Sbrana S, Azzara A, Minnucci S, Angiolini C (1998). Detection of eosinophils in whole blood samples by flow cytometry.. Cytometry.

[32] Fujiwara RT, Geiger SM, Bethony J, Mendez S (2006). Comparative immunology of human and animal models of hookworm infection.. Parasite Immunol.

[33] Teixeira-Carvalho A, Fujiwara RT, Stemmy E, Olive D, Damsker JM (2008). Binding of excreted/secreted products of adult Necator americanus to human NK cells in hookworm infected individuals from Brazil.. Infect Immun.

[34] Hsieh GC, Loukas A, Wahl AM, Bhatia M, Wang Y (2004). A secreted protein from the human hookworm necator americanus binds selectively to NK cells and induces IFN-gamma production.. J Immunol.

[35] Culley FJ, Brown A, Conroy DM, Sabroe I, Pritchard DI (2000). Eotaxin is specifically cleaved by hookworm metalloproteases preventing its action in vitro and in vivo.. J Immunol.

[36] Hartgers FC, Obeng BB, Kruize YC, Duijvestein M, de Breij A (2008). Lower Expression of TLR2 and SOCS-3 Is Associated with Schistosoma haematobium Infection and with Lower Risk for Allergic Reactivity in Children Living in a Rural Area in Ghana.. PLoS Negl Trop Dis.

[37] Babu S, Blauvelt CP, Kumaraswami V, Nutman TB (2006). Cutting edge: diminished T cell TLR expression and function modulates the immune response in human filarial infection.. J Immunol.

[38] Leon B, Lopez-Bravo M, Ardavin C (2005). Monocyte-derived dendritic cells.. Semin Immunol.

[39] Matyszak MK, Citterio S, Rescigno M, Ricciardi-Castagnoli P (2000). Differential effects of corticosteroids during different stages of dendritic cell maturation.. Eur J Immunol.

[40] Zhang R, Bharadwaj U, Li M, Chen C, Yao Q (2007). Effects of pentoxifylline on differentiation, maturation, and function of human CD14+ monocyte-derived dendritic cells.. J Immunother.

[41] McRae BL, Nagai T, Semnani RT, van Seventer JM, van Seventer GA (2000). Interferon-alpha and -beta inhibit the in vitro differentiation of immunocompetent human dendritic cells from CD14(+) precursors.. Blood.

[42] Bueno LL, Fujiwara RT, Soares IS, Braga EM (2008). Direct effect of Plasmodium vivax recombinant vaccine candidates AMA-1 and MSP-119 on the innate immune response.. Vaccine.

[43] Urban BC, Ferguson DJ, Pain A, Willcox N, Plebanski M (1999). Plasmodium falciparum-infected erythrocytes modulate the maturation of dendritic cells.. Nature.

[44] MacDonald AS, Straw AD, Bauman B, Pearce EJ (2001). CD8- dendritic cell activation status plays an integral role in influencing Th2 response development.. J Immunol.

[45] Maxwell C, Hussain R, Nutman TB, Poindexter RW, Little MD (1987). The clinical and immunologic responses of normal human volunteers to low dose hookworm (Necator americanus) infection.. Am J Trop Med Hyg.

[46] White CJ, Maxwell CJ, Gallin JI (1986). Changes in the structural and functional properties of human eosinophils during experimental hookworm infection.. J Infect Dis.

[47] Padigel UM, Lee JJ, Nolan TJ, Schad GA, Abraham D (2006). Eosinophils can function as antigen-presenting cells to induce primary and secondary immune responses to Strongyloides stercoralis.. Infect Immun.

[48] Linsley PS, Golstein P (1996). Lymphocyte activation: T-cell regulation by CTLA-4.. Curr Biol.

[49] Shi HZ (2004). Eosinophils function as antigen-presenting cells.. J Leukoc Biol.

[50] Lotfi R, Lotze MT (2008). Eosinophils induce DC maturation, regulating immunity.. J Leukoc Biol.

[51] Zimpfer U, Dichmann S, Termeer CC, Simon JC, Schroder JM (2000). Human dendritic cells are a physiological source of the chemotactic arachidonic acid metabolite 5-oxo-eicosatetraenoic acid.. Inflamm Res.

[52] Corthay A (2006). A three-cell model for activation of naive T helper cells.. Scand J Immunol.

[53] Jutel M, Blaser K, Akdis CA (2005). Histamine in allergic inflammation and immune modulation.. Int Arch Allergy Immunol.

[54] Caron G, Delneste Y, Roelandts E, Duez C, Herbault N (2001). Histamine induces CD86 expression and chemokine production by human immature dendritic cells.. J Immunol.

[55] Elenkov IJ, Webster E, Papanicolaou DA, Fleisher TA, Chrousos GP (1998). Histamine potently suppresses human IL-12 and stimulates IL-10 production via H2 receptors.. J Immunol.

[56] Lambrecht BN, Hammad H (2003). Taking our breath away: dendritic cells in the pathogenesis of asthma.. Nat Rev Immunol.

[57] Lambrecht BN, Salomon B, Klatzmann D, Pauwels RA (1998). Dendritic cells are required for the development of chronic eosinophilic airway inflammation in response to inhaled antigen in sensitized mice.. J Immunol.

[58] Loukas A, Constant SL, Bethony JM (2005). Immunobiology of hookworm infection.. FEMS Immunol Med Microbiol.

